# Mild thermal unfolding of pea protein enhances curcumin binding and interfacial properties of the resulting complexes

**DOI:** 10.1016/j.fochx.2026.103815

**Published:** 2026-04-05

**Authors:** Lijuan Luo, Ping Li, Hui Yang, Mingwei Zhang

**Affiliations:** a Institute of Quality Standard and Monitoring Technology for Agro-products of Guangdong Academy of Agricultural Sciences, Guangzhou 510640, China; b Sericultural & Agri-Food Research Institute Guangdong Academy of Agricultural Sciences, Key Laboratory of Functional Foods, Ministry of Agriculture and Rural Affairs, Guangdong Key Laboratory of Agricultural Products Processing, Guangzhou 510610, China; c Guangdong Provincial Key Laboratory of Quality & Safety Risk Assessment for Agro-products, Guangzhou 510640, China

**Keywords:** Thermal modification, Curcumin encapsulation, Protein-polyphenol interactions, Interfacial properties

## Abstract

This study investigated how thermal treatment (80–100 °C) alters the structure of pea protein isolate (PPI) and affects curcumin encapsulation and the interfacial properties of PPI-curcumin complexes. Heating induced progressive tertiary loosening, as shown by fluorescence red shifts, increased surface hydrophobicity, and more negative zeta potential. Treatment at 90 °C produced the most favorable structural state, yielding the highest encapsulation efficiency and loading capacity and generating smaller, more uniform nanoparticles with improved stability. FTIR and XRD confirmed that curcumin was incorporated in an amorphous form and associated with PPI mainly through hydrogen bonding and hydrophobic interactions. Fluorescence quenching revealed stronger static binding in the heated samples, particularly at 90 °C. Interfacial tension and dilatational rheology showed that H90 and its complexes formed the most cohesive and elastic interfacial films. Overall, moderate thermal unfolding enhanced curcumin binding and interfacial functionality, supporting the design of heat-modified plant protein carriers in emulsion systems.

## Highlights

• Thermal treatment improved PPI structural flexibility and hydrophobic exposure.
• Heating at 90 °C maximized curcumin encapsulation and binding affinity.
• Heat-treated PPI formed smaller and more uniform curcumin-loaded nanoparticles.
• H90 and H90–Cur produced the strongest and most elastic interfacial films.

## 1 Introduction

The enhancement of nutraceutical delivery and bioavailability has become a central objective in functional food development, driven by growing consumer demand for health-promoting dietary components (Guan et al., 2025). Polyphenols, such as curcumin, represent a major category of plant-derived bioactives known for their potent antioxidant, anti-inflammatory, and therapeutic properties (Davinelli et al., 2025). However, their practical application is constrained by intrinsic limitations, including extremely low water solubility, poor dispersibility, and chemical instability, which collectively restrict gastrointestinal absorption and diminish biological efficacy (Liu et al., 2023). Protein-based nanocarriers have emerged as an effective strategy to overcome these challenges by encapsulating hydrophobic compounds, thereby improving their stability and apparent solubility. Moreover, the functional performance of protein carriers can be further enhanced through tailored physical or chemical modifications, making carrier engineering a critical dimension in the design of nutraceutical delivery systems (Ji et al., 2023).

The selection of an appropriate protein is essential for developing efficient, safe, and consumer-acceptable nanocarriers. Plant proteins have gained increasing attention as sustainable, biocompatible, and structurally versatile materials (Sarathy et al., 2025). Among them, pea protein isolate (PPI) offers advantages such as balanced amino acid composition and low allergenicity (Kim et al., 2025). Nevertheless, native PPI possesses a relatively rigid tertiary structure, limited molecular flexibility, and strong intermolecular associations, resulting in low surface hydrophobicity and restricted affinity toward hydrophobic bioactives (Asen & Aluko, 2022). These inherent properties also limit its ability to form cohesive interfacial films, thereby constraining its broader application as an efficient carrier in emulsion-based delivery systems.

Thermal treatment is widely used in food processing due to its operational simplicity, controllability, and cost-effectiveness, and it provides a practical means of modifying protein structure (Chen et al., 2025). Controlled heating can induce varying degrees of unfolding, aggregation, and reorganization of molecular flexibility and hydrophobic/hydrophilic balance, thereby improving surface functionality and enhancing protein performance in delivery applications (Guo et al., 2024). Because protein-polyphenol interactions are largely entropy-driven, heat-induced exposure of hydrophobic residues generally increases binding affinity (Zhang, Cheng, et al., 2021). For example, Cao and Xiong (2017) reported that heat-unfolded whey protein isolate exhibits increased binding sites for gallic acid and epigallocatechin gallate compared to unheated whey protein. Zhong et al. (2024) also reported that moderate heating (85 °C) promoted the opening of the hydrophobic cavity in β-lactoglobulin, strengthening both hydrogen bonding and hydrophobic interactions with theaflavin-3,3′-digallate. However, excessive heating may also cause irreversible denaturation, extensive aggregation, and loss of functionality (Wu et al., 2026). Importantly, despite growing interest in protein-polyphenol systems, how thermal denaturation modulates the surface properties of PPI and subsequently influences its delivery capability and complex formation remains largely unexplored.

In addition to improving binding capacity, the interfacial behavior of protein-polyphenol complexes plays a critical role in determining the stability and performance of emulsion-based delivery systems. Protein structural unfolding and ligand binding can profoundly alter interfacial adsorption kinetics, interfacial film elasticity, and the overall stabilization of oil-water interfaces (Chen et al., 2024). Previous studies on heat-treated pea protein have mainly focused on emulsifying properties, while studies on protein-polyphenol systems have primarily addressed binding behavior or delivery performance. However, how heat-induced structural changes in PPI affect both curcumin complexation and the interfacial functionality of the resulting complexes remains unclear.

Therefore, the objective of this study was to investigate the effects of thermal treatment (80–100 °C) on pea protein isolate (PPI) structure and its subsequent impact on curcumin encapsulation and interfacial properties of PPI-curcumin complexes. By integrating fluorescence spectroscopy, hydrophobicity analysis, FTIR, XRD, fluorescence quenching, and interfacial rheology, this work establishes a comprehensive mechanistic understanding of how controlled thermal unfolding alters PPI’s interaction capacity and interfacial performance in protein-curcumin complexes. These insights provide valuable guidance for designing heat-modified plant proteins as effective carriers for hydrophobic nutraceuticals and as functional emulsifiers in food systems.

## 2 Materials and methods

### 2.1 Materials

Yellow pea seeds (*Pisum sativum* L.) were sourced from Yantai Shuangta Food Co., Ltd. (Yantai, Shandong, China). Soybean oil, obtained from a local supermarket, was purified by mixing with florisil (60–100 mesh, 5% w/w) and stirring overnight at room temperature (25–27 °C) to remove surface-active impurities. The purified oil was collected after centrifugation at 10,000 *g* for 20 min. Curcumin (94% purity) and 8-anilino-1-naphthalenesulfonic acid ammonium salt hydrate (ANS) were from Sigma-Aldrich (Shanghai, China). All reagents were from Sinopharm Chemical Reagent Co., Ltd. (Shanghai, China). Deionized water was used in all experiments.

### 2.2 Preparation of PPI and HPPI

Pea protein was prepared using an alkali extraction-acid precipitation method as previously described (Luo et al., 2023). Briefly, defatted pea flour was dispersed in distilled water (1:10, w/v), adjusted to pH 8.0, and stirred for 2 h at room temperature. After centrifugation, the supernatant was adjusted to pH 4.5 for protein precipitation. The precipitate was washed, redissolved in water, adjusted to pH 7.0, dialyzed against distilled water, and then freeze-dried. The final protein content of the obtained PPI was 86.60% (w/w, dry basis). The freeze-dried PPI powder was dissolved in PBS (pH 7, 10 mM) to obtain a 3% solution, which was stirred at room temperature for 2 h to ensure complete dissolution. Equal portions of the PPI solution were placed in sealed bottles and heated at 80 °C, 90 °C, and 100 °C with stirring for 20 min to obtain thermally treated PPI (HPPI) samples, denoted as H80, H90, and H100, respectively. These temperatures were selected based on the denaturation temperature of pea protein (approximately 75 °C) and their relevance to food processing.

### 2.3 Characterization of HPPI

#### 2.3.1 Intrinsic fluorescence spectroscopy

PPI and HPPI were dissolved in PBS (pH 7.0, 10 mM) to 0.2 mg/mL. Intrinsic fluorescence spectra were recorded using a fluorescence spectrometer (F7000, Hitachi, Tokyo, Japan) with excitation at 280 nm and emission measured from 300 to 400 nm, with both excitation and emission slit widths set to 5 nm.

#### 2.3.2 Surface hydrophobicity

Surface hydrophobicity was determined using the method of Kato and Nakai (1980). PPI and HPPI were prepared in PBS (pH 7.0, 10 mM) at concentrations ranging from 0.04 to 0.2 mg/mL. A 10 μL aliquot of ANS solution (8 mmol/L in PBS) was added to 1 mL of the protein solution. Fluorescence intensity was measured at excitation 390 nm and emission 470 nm using an Infinite M200pro microplate reader (Tecan, Switzerland). A mixture without ANS (replaced by PBS) served as the blank. The initial slope of the fluorescence intensity versus protein concentration plot represented the surface hydrophobicity (H_0_) of the protein.

### 2.4 Preparation of protein-curcumin nanoparticles

The nanoparticles were prepared using the anti-solvent precipitation method (Liu et al., 2017). PPI and HPPI were diluted to 1 mg/mL with 10 mM PBS. Curcumin was dissolved in anhydrous ethanol to obtain a 10 mM stock solution, within its solubility in ethanol. Under stirring, 100 μL of the curcumin stock solution was slowly added to 2.9 mL of the pea protein solution. After stirring in the dark at room temperature for 30 min, the mixture was centrifuged at 10,000 *g* for 20 min. The pea protein-curcumin nanoparticles were collected from the supernatant, denoted as NPPI-Cur, H80-Cur, H90-Cur, and H100-Cur, and a portion of each nanoparticle suspension was lyophilized for further analysis.

### 2.5 Encapsulation efficiency and loading capacity

The encapsulated curcumin was extracted from the nanoparticles using ethanol (Yuan et al., 2021). The supernatant obtained in Section 2.3 was mixed with ethanol at a 1:19 (v/v) ratio, vortexed for 5 min, and then centrifuged at 10,000 *g* for 20 min. The curcumin concentration in the supernatant was determined at 426 nm using a microplate reader and quantified using a standard curve of free curcumin in 95% ethanol (A = 2.41685C, R^2^ = 0.9999), where C is the curcumin concentration in mM. The encapsulation efficiency (EE) and loading capacity (LC) of curcumin were calculated using the following equations:

$$\mathrm{EE}\;\left(\%\right)=\left[\text{Encapsulated curcumin}\;\left(\mathrm{mg}\right)/\text{Total curcumin}\;\left(\mathrm{mg}\right)\right]\times 100$$

$$\mathrm{LC}\;\left(\%\right)=\left[\text{Encapsulated curcumin}\;\left(\mathrm{mg}\right)/\text{Total mass of nanoparticles}\;\left(\mathrm{mg}\right)\right]\times 100$$

### 2.6 Particle characterization

#### 2.6.1 Size and zeta potential

The average particle size and zeta potential of the nanoparticles were determined by dynamic light scattering (DLS). Samples were diluted to 0.02% (w/v) in PBS (pH 7.0, 10 mM) and measured using a Nano-ZS Zetasizer (Malvern Instruments, Worcestershire, UK) at 25 °C.

#### 2.6.2 Morphology

The morphology of the blank and curcumin-loaded nanoparticles was examined using field emission scanning electron microscopy (FE-SEM). The freshly lyophilized powders were coated with a gold layer using a sputter coater (EMS 150 T, England) and imaged on a Zeiss Merlin FE-SEM (Carl Zeiss Microscopy GmbH, Germany) at an accelerating voltage of 5 kV.

### 2.7 FTIR and XRD spectroscopy

The FTIR spectra of the freeze-dried powders of PPI, HPPI, curcumin, physical mixtures, and curcumin-loaded nanoparticles were recorded using an FTIR spectrometer (IRTracer-100, Shimadzu Co., Kyoto, Japan) in the range of 4000–400 cm^−1^ with a resolution of 4 cm^−1^.

XRD patterns of the samples were collected using an X-ray diffractometer (D8 Advance, Bruker Corporation, Germany) at an accelerating voltage of 40 kV and a current of 40 mA. The scanning speed was 0.1 s/step, with a range of 5° to 40° and a step size of 0.02°.

### 2.8 Fluorescence quenching

The binding characteristics of curcumin with protein were assessed by fluorescence quenching (Ji et al., 2023). PPI and HPPI were dissolved in PBS (pH 7.0, 10 mM) to a concentration of 0.2 mg/mL. A 980 μL aliquot of the protein solution was mixed with 0–20 μL of a 10 mM curcumin ethanol solution, and the volume was adjusted to 1 mL with ethanol, resulting in curcumin concentrations ranging from 0 to 20 μM. Fluorescence emission spectra were recorded over the range of 300–450 nm with an excitation wavelength of 280 nm. The intrinsic fluorescence spectra of the protein were measured to evaluate the quenching effect of curcumin and the binding characteristics between curcumin and the protein. To correct for the inner filter effect, Eq. (1) was used as follows:

(1) $$F_{c}=F_{m}10^{\left(A_{\mathrm{ex}}+A_{\mathrm{em}}\right)/2}$$

where F_c_ and F_m_ represent the fluorescence intensities after correction and measurement, respectively, and A_ex_ and A_em_ are the absorbances of curcumin at the excitation and emission wavelengths, respectively.

Fluorescence quenching mechanisms were analyzed using the Stern-Volmer Eq. (2). The binding constant (Ka) and number of binding sites (n) were calculated using a double logarithmic equation based on fluorescence data (3).

(2) $$\frac{F_{0}}{F}=1+K_{\mathrm{sv}}\times \left[\mathrm{Cur}\right]=1+K_{q}\times \tau_{0}\times \left[\mathrm{Cur}\right]$$

(3) $$\log\;\left[\left(F_{0}-F\right)/F\right]=\log\;K_{a}+\mathrm{nlog}\;\left[\mathrm{Cur}\right]$$

where *F*_0_ is the initial fluorescence intensity of the protein (including PPI and HPPI), and F is the corrected fluorescence intensity of the protein in the presence of curcumin. Ksv represents the Stern-Volmer quenching constant. [Cur] denotes the curcumin concentration. Kq is the quenching rate constant, and τ_0_ is the fluorophore lifetime (10^−8^ s for most biomolecules). Ka is the binding constant between protein and curcumin, and n refers to the number of binding sites.

### 2.9 Interfacial tension

Interfacial tension was measured using the pendant drop method, as described in our previous study (Luo et al., 2023). The interfacial properties of samples at the oil-water interface were assessed at 25 °C using an optical contact angle meter (OCA25, DataPhysics Instruments GmbH, Germany) equipped with an oscillating drop accessory (ODG-20). A 20 μL drop of the protein sample (1 mg/mL) was suspended at the tip of a needle immersed in purified soybean oil inside an optical glass cuvette. The drop was captured by a camera over a 180-min period, and its contour was analyzed by fitting the data to the Young-Laplace equation to calculate the interfacial tension. The densities of the protein solution (in PBS) and the oil were 0.9982 g/mL and 0.9142 g/mL, respectively.

### 2.10 Dilatational rheological properties

Following the interfacial tension measurements, frequency sweeps were performed on the droplet using the same instrument. The frequency sweep was conducted at frequencies ranging from 0.005 to 0.1 Hz with a constant amplitude of 5%. Each sweep consisted of 5 cycles, followed by a 300-s rest period. The oscillating surface tension signals were analyzed using a fast Fourier transform to calculate the complex surface dilatational modulus (E*), the dilatational elastic modulus (E′), and the dilatational viscous modulus (E″).

### 2.11 Data analysis

Unless otherwise specified, all assays were performed in triplicate, and the results are expressed as mean ± standard deviation. Data were analyzed by one-way ANOVA, followed by Duncan’s post hoc test for significance, with a *p*-value <0.05 considered statistically significant. Statistical analysis was conducted using SPSS software.

## 3 Results and discussion

### 3.1 Intrinsic fluorescence spectroscopy

**Fig. 1:**
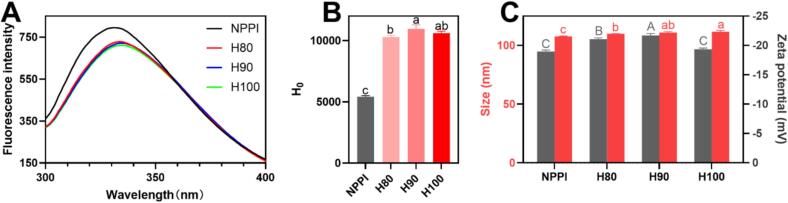
Intrinsic fluorescence spectra (A), surface hydrophobicity (B), and zeta potential and particle size (C) of pea protein isolate before and after thermal treatment.

The intrinsic fluorescence spectra of PPI before and after heat treatment were measured to observe changes in the protein’s conformation. The intrinsic fluorescence spectra of pea protein isolates were significantly altered by heat treatment, as shown in Fig. 1A. Heating caused a progressive red shift of the maximum emission wavelength from 331 nm (NPPI) to 334 nm (H80 and H100) and 335 nm (H90), accompanied by a gradual decrease in fluorescence intensity (from 795 for NPPI to 728, 721, and 711 for H80, H90, and H100, respectively). The red shift indicates that heating induced partial unfolding of the protein, which exposed previously buried tryptophan residues to the solvent. Among the heat-treated groups, H90 displayed the greatest red shift (335 nm), suggesting that 90 °C resulted in the most significant structural unfolding and exposure of chromophoric residues. The H100 sample also exhibited a red shift but showed a less pronounced shift (334 nm), coupled with the strongest fluorescence quenching, suggesting that further heating led to aggregation-driven quenching, which partially reburied fluorophores. This may be attributed to further exposure of hydrophobic groups at higher temperature, which enhanced intermolecular hydrophobic interactions and promoted aggregation. Similar intrinsic fluorescence changes after thermal denaturation have been reported for other plant proteins such as *Moringa oleifera* lam. Leaf protein (Xi et al., 2025) and quinoa protein (He et al., 2022). Thus, the data suggest that 90 °C is the optimal temperature for maximal exposure of fluorophores without excessive aggregation, which is critical for the subsequent interactions with hydrophobic compounds such as curcumin.

### 3.2 Surface hydrophobicity

Surface hydrophobicity was measured to evaluate the surface properties induced by heating. As shown in Fig. 1B, all heat-treated pea protein samples exhibited a significant increase in surface hydrophobicity compared to the NPPI. The surface hydrophobicity values for NPPI, H80, H90, and H100 were 5424 ± 88, 10293 ± 149, 10965 ± 331, and 10613 ± 166, respectively. This substantial increase reflects the exposure of buried hydrophobic residues caused by partial unfolding of the protein matrix during heating. Among the heat-treated groups, H90 exhibited the highest surface hydrophobicity, which was consistent with its most significant red shift in fluorescence, confirming that this treatment induced the maximum exposure of hydrophobic patches. Although H100 also showed elevated hydrophobicity, its slightly lower H₀ value compared with H90 likely results from the onset of aggregation at higher temperatures, which can partially re-bury hydrophobic regions and reduce measurable surface exposure (Wang et al., 2016). The parallel increases in fluorescence red shift and H₀ confirm that controlled thermal unfolding effectively modulates the structural flexibility of PPI and creates a more hydrophobic, interaction-ready protein surface.

### 3.3 Zeta potential and size

The zeta potential and particle size of the native and heat-treated pea protein isolates are shown in Fig. 1C. The zeta potential of NPPI was −19.0 ± 0.3 mV, which became more negative after heating to −21.1 ± 0.2 mV (H80) and − 21.7 ± 0.4 mV (H90), indicating increased surface charge due to thermal unfolding and exposure of ionizable groups. This enhanced charge density suggests improved colloidal stability for H80 and H90 (Dahatonde et al., 2025). In contrast, H100 exhibited a less negative value (−19.4 ± 0.2 mV), implying that excessive heating promoted partial aggregation, thereby reducing the effective surface charge available for electrostatic stabilization.

Particle size increased slightly from 107.6 ± 0.5 nm (NPPI) to 109.9 ± 0.2 nm (H80), 111.0 ± 0.8 nm (H90), and 111.7 ± 0.9 nm (H100). This gradual enlargement indicates thermally induced protein-protein association (Peng et al., 2016). The largest size observed for H100 is consistent with its reduced zeta potential, reflecting aggregation at elevated temperatures. Together, these results show that H90 offers an optimal balance between increased surface charge and controlled aggregation, producing a structurally flexible yet colloidally stable protein system, consistent with its higher hydrophobic exposure and moderate unfolding observed in fluorescence and hydrophobicity analyses.

### 3.4 Encapsulation efficiency and loading capacity

The EE and LC of curcumin in PPI and HPPI are summarized in Table 1. Both indices increased significantly after thermal treatment. EE increased from 72.1 ± 0.4% for NPPI to 85.2 ± 2.0% for H80, 89.5 ± 1.7% for H90, and 85.8 ± 1.0% for H100. Similarly, LC rose from 26.6 ± 0.1% to 31.4 ± 0.7%, 33.0 ± 0.6%, and 31.6 ± 0.4%, respectively. Such high loading levels exceed those of many reported protein systems, including soy protein (≈12%) and whey protein (≈18%) when binding curcumin (Peng et al., 2020). The enhancement of EE and LC in all heated samples results from heat-induced unfolding, which exposes hydrophobic cavities and aromatic residues that favor curcumin binding (Wei et al., 2021). Among the treatments, H90 exhibited the highest EE and LC, indicating that heating to 90 °C produced an optimally expanded protein conformation with maximally accessible hydrophobic regions. Although H100 also enhanced encapsulation relative to NPPI, its lower performance compared with H90 suggests that excessive heating led to aggregation through hydrophobic and disulfide interactions, reducing the number of available binding sites (Horta et al., 2025). Overall, these results indicate that H90, with the highest surface hydrophobicity, had more exposed hydrophobic groups available for interaction with curcumin, which contributed to its highest encapsulation efficiency and loading capacity.

**Table 1:** Encapsulation efficiency, loading capacity, particle size, and zeta potential of curcumin-loaded pea protein prepared from native and heat-treated PPI.

Sample	EE (%)	LC (%)	Size (nm)	ζ potential (mV)
NPPI	72.1 ± 0.4^c^	26.6 ± 0.1^c^	109.6 ± 0.5^a^	−26.3 ± 0.3^a^
H80	85.2 ± 2.0^b^	31.4 ± 0.7^b^	98.2 ± 0.7^b^	−24.8 ± 0.4^bc^
H90	89.5 ± 1.7^a^	33.0 ± 0.6^a^	98.9 ± 1.0^b^	−25.2 ± 0.5^b^
H100	85.8 ± 1.0^b^	31.6 ± 0.4^b^	98.1 ± 0.2^b^	−24.4 ± 0.4^c^

Notes: EE, encapsulation efficiency; LC, loading capacity. Values are expressed as mean ± standard deviation. Different letters within the same column indicate significant differences (*p* < 0.05).

### 3.5 Zeta potential and particle size of protein-curcumin nanoparticles

The incorporation of curcumin altered both the surface charge and particle size of the resulting protein nanoparticles (Table 1). Compared with their parent proteins, all PPI-Cur nanoparticles exhibited more negative zeta potentials, increasing from −19.0 mV (NPPI) to −26.3 mV (NPPI-Cur) and from −21.1 to −24.8 mV (H80-Cur), −21.7 to −25.2 mV (H90-Cur), and − 19.4 to −24.4 mV (H100-Cur). The increased surface charge suggests that curcumin binding alters the distribution of charged groups on the protein surface and improves colloidal stability through stronger electrostatic repulsion.

Curcumin loading also influenced particle size. NPPI-Cur NPs exhibited a slight size increase (from 107.6 to 109.6 nm), consistent with the more rigid conformation of NPPI and its tendency to form looser aggregates upon curcumin binding. In contrast, HPPI-Cur nanoparticles were significantly smaller than their corresponding proteins (from ∼111 nm to 98.1–98.9 nm). This reduction is attributed to the greater structural flexibility of heat-treated proteins, which facilitates more compact packing around curcumin and yields smaller, more uniform nanoparticles (Zhang, Dong, et al., 2021). These results align with the higher surface charge observed for HPPI-Cur samples, suggesting enhanced electrostatic repulsion that helps prevent aggregation and maintains nanoscale uniformity.

Collectively, the zeta potential and size analyses indicate that moderate thermal unfolding (particularly at 90 °C) enables the formation of highly stable, compact PPI-Cur nanoparticles, while the rigid structure of NPPI limits its ability to reorganize upon curcumin incorporation.

### 3.6 Morphological characteristics of protein-curcumin nanoparticles

**Fig. 2:**
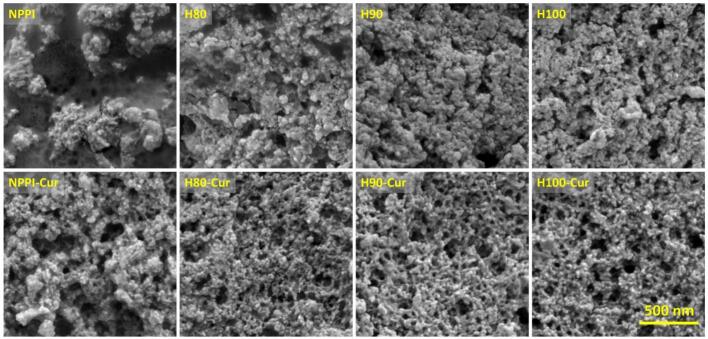
Field-emission scanning electron micrographs of native and heat-treated pea protein isolates and their corresponding PPI–curcumin complexes.

The morphological features of PPI and protein-curcumin nanoparticles observed by FE-SEM are presented in Fig. 2. After freeze-drying, all samples exhibited aggregated or network-like appearances, which is commonly attributed to particle association during water removal. At the single-particle level, the observed particles were considerably smaller than the hydrodynamic diameters measured by DLS, which can be attributed to the absence of the hydration layer and shrinkage during lyophilization (Sha et al., 2025).

Among the protein samples, NPPI formed a relatively compact aggregated structure, consistent with its more rigid conformation and limited flexibility. In contrast, the heat-treated proteins displayed more open and continuous network-like structures, particularly in H90 and H100, reflecting their greater structural mobility and higher propensity for intermolecular association rather than random aggregation during freeze-drying. The resulting network structures of HPPI indicate a balance between molecular flexibility and interparticle interaction, which agrees with their improved colloidal stability observed from zeta potential measurements.

Curcumin incorporation further intensified the network-like morphology in both NPPI-Cur and HPPI-Cur samples. NPPI-Cur exhibited networks with larger and more irregular pores, reflecting its stronger tendency toward aggregation. In contrast, HPPI-Cur nanoparticles formed denser and more uniform networks with smaller pores, aligned with their smaller particle sizes and more negative zeta potentials. At the single-particle level, NPPI-Cur NPs showed greater aggregation and larger particle sizes, while HPPI-Cur NPs exhibited smaller and more homogeneous particles, consistent with DLS measurements. Notably, the HPPI-Cur NPs were smaller than HPPI before encapsulation, consistent with DLS results, suggesting that the enhanced structural flexibility and surface hydrophobicity of HPPI facilitate tighter curcumin-protein packing, while the higher surface charge further stabilizes the nanoparticles and minimizes aggregation during drying (Lin et al., 2026). Overall, these morphological observations support earlier structural findings: heat treatment enhances protein flexibility and dispersibility, enabling the formation of compact and uniform curcumin-loaded nanoparticles, while the rigid NPPI structure leads to larger, less uniform assemblies.

### 3.7 FTIR spectra and secondary structure

**Fig. 3:**
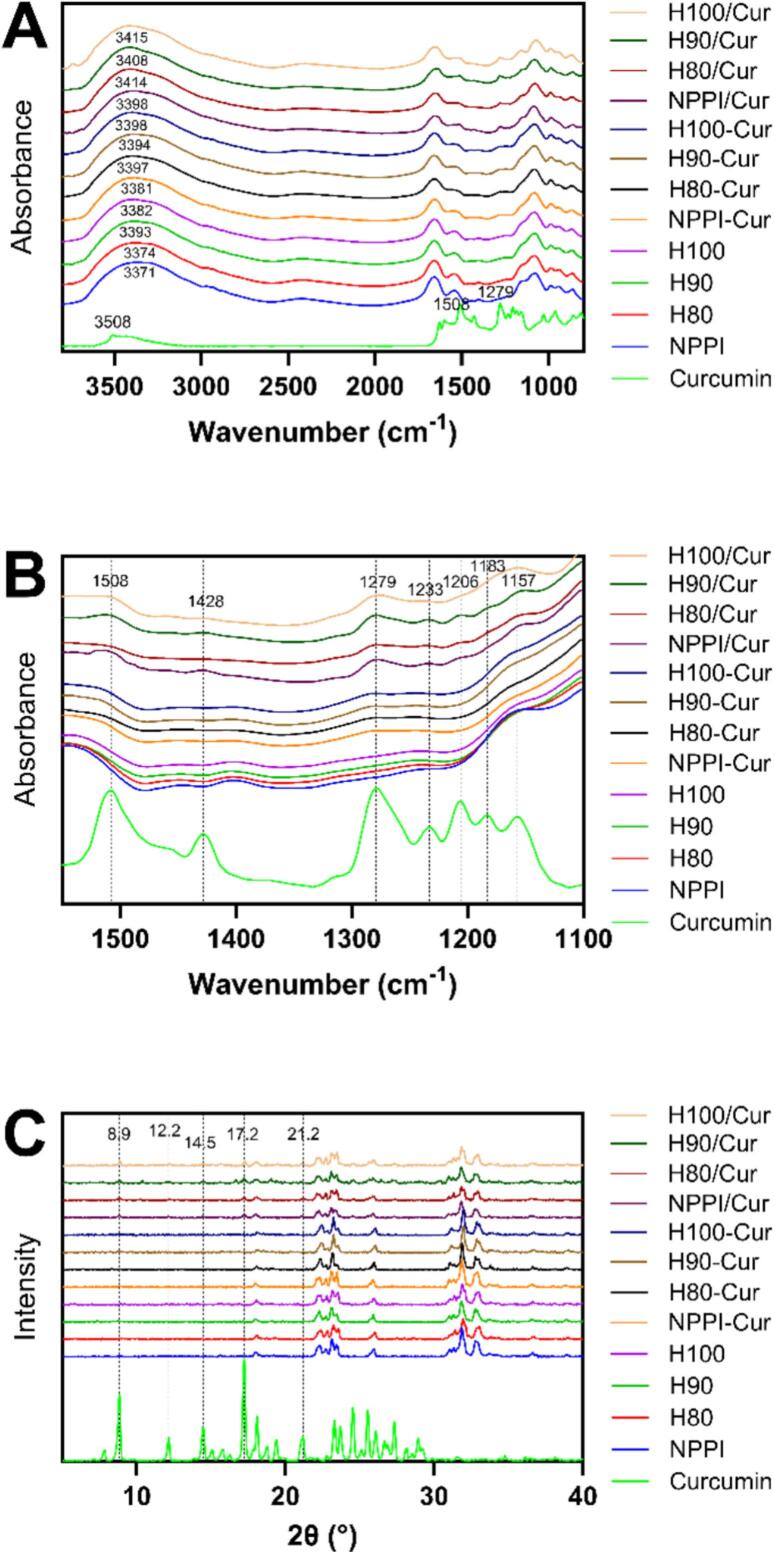
FTIR spectra of free curcumin, native and heat-treated pea protein isolates, physical mixtures (protein/Cur), and PPI–curcumin complexes: full-range spectra (A) and expanded spectra in the 1550–1100 cm^−1^ region (B); and (C) XRD patterns of the same samples.

The FTIR spectra of PPI before and after heating and their complexes with curcumin are shown in Fig. 3A. Heating caused the amide A band of NPPI at 3371 cm^−1^ to shift to 3374, 3393, and 3382 cm^−1^ for H80–H100, indicating weakening of protein-protein hydrogen bonding and partial exposure of N–H/O–H groups as the tertiary structure loosened (Badjona et al., 2024). Minor shifts were also observed in several side-chain vibration regions, including 1446 → 1448 cm^−1^, 1402 → 1402–1404 cm^−1^, and 1079 → 1079–1081 cm^−1^, indicating subtle rearrangements of noncovalent interactions upon heating. The amide I (1658 cm^−1^) and amide II (1545 cm^−1^) peaks remained almost unchanged, suggesting that thermal treatment primarily affected supramolecular packing rather than the peptide backbone.

After curcumin incorporation, the amide A peak shifted further to 3381–3398 cm^−1^ in all protein-Cur complexes, suggesting that curcumin binding altered the local hydrogen-bonding environment of the protein. Notably, the characteristic curcumin peaks—1508, 1428, 1279, 1233, 1206, 1183 and 1157 cm^−1^—were present in the physical mixtures (Fig. 3B), but absent in the spectra of all protein-Cur complexes. This indicates that curcumin was not present in a free form, but was incorporated within the protein matrix, similar to the previous study using ovalbumin, casein and soy proteins to load curcumin (Feng et al., 2019; Pan et al., 2013; Yuan et al., 2021). Minor shifts in the amide I and II bands upon curcumin binding (e.g., 1656 → 1657–1658 cm^−1^; 1547 → 1547–1550 cm^−1^) suggest that hydrogen bonding and hydrophobic interactions were involved in stabilizing the complexes.

The secondary structure results derived from amide I deconvolution (Table 2) further clarify the effect of heating and curcumin loading. Heating caused a clear increase in intermolecular β-sheet content (from 20.81% → 21.66%, 21.63%, and 22.38% for H80, H90, and H100), indicating that moderate thermal treatment induced partial unfolding of PPI and promoted rearrangement of intermolecular hydrogen bonds, thereby favoring β-sheet-mediated association (Luo et al., 2023). Simultaneously, β-turn content decreased (25.16% → 24.44%), suggesting reduced local flexibility. Random coil, α-helix, and intramolecular β-sheet structures remained largely unchanged, demonstrating that heating primarily reorganized intermolecular packing rather than altering intrinsic secondary motifs.

**Table 2:** Secondary structure composition of PPI before and after heating and their complexes with curcumin.

Sample	Intermolecular β-sheet	Random coil	α-helix	β-turn	Intramolecular β-sheet
NPPI	20.81 ± 0.01%^d^	17.77 ± 0.02%^bc^	18.30 ± 0.01%^a^	25.16 ± 0.01%^ab^	17.96 ± 0.00%^ab^
H80	21.66 ± 0.06%^bc^	17.49 ± 0.01%^c^	18.24 ± 0.00%^a^	25.12 ± 0.07%^ab^	17.48 ± 0.02%^c^
H90	21.63 ± 0.11%^c^	17.75 ± 0.02%^bc^	18.29 ± 0.10%^a^	24.68 ± 0.01%^bc^	17.64 ± 0.02%^bc^
H100	22.38 ± 0.06%^a^	17.43 ± 0.01%^c^	18.16 ± 0.03%^a^	24.44 ± 0.03%^cd^	17.60 ± 0.01%^bc^
NPPI-Cur	20.33 ± 0.14%^e^	18.39 ± 0.04%^a^	18.48 ± 0.05%^a^	24.72 ± 0.07%^bc^	18.08 ± 0.02%^a^
H80-Cur	20.89 ± 0.15%^d^	18.05 ± 0.08%^ab^	18.39 ± 0.19%^a^	24.85 ± 0.22%^abc^	17.82 ± 0.19%^abc^
H90-Cur	20.79 ± 0.19%^d^	17.84 ± 0.04%^bc^	18.44 ± 0.11%^a^	25.42 ± 0.08%^a^	17.51 ± 0.04%^c^
H100-Cur	22.02 ± 0.04%^b^	18.16 ± 0.37%^ab^	18.16 ± 0.17%^a^	23.87 ± 0.47%^d^	17.79 ± 0.23%^abc^

Note: Values represent mean ± standard deviation. Different letters within the same column indicate significant differences (*p* < 0.05).

Curcumin loading produced a different pattern. The intermolecular β-sheet content decreased slightly in all complexes, indicating that curcumin partially interfered with protein-protein alignment. In parallel, random coil content increased, reflecting localized structural relaxation upon curcumin insertion. β-turn generally decreased upon binding; however, H90-Cur displayed a notable increase (24.68% → 25.42%), indicating that the moderately unfolded H90 structure retained higher adaptability for local rearrangement upon curcumin binding. The α-helix and intramolecular β-sheet contents remained essentially unchanged, showing that curcumin primarily affected flexible regions rather than intrinsic backbone motifs.

Overall, the FTIR and secondary-structure results suggest that controlled thermal unfolding enhances hydrophobic and hydrogen-bonding accessibility, while curcumin incorporation induces localized rearrangements without disrupting the protein backbone. These structural transitions support the superior encapsulation and nanoparticle formation observed particularly in H90.

### 3.8 XRD

The XRD patterns of PPI, curcumin, their physical mixtures, and the PPI-Cur complexes are shown in Fig. 3C. Native and heat-treated PPI exhibited several broad diffraction peaks at 17.98°, 22.3°, 22.7°, 23.1°, 23.4°, 26.0°, 31.0°, 31.3°, 31.9°, and 32.9°, consistent with the largely amorphous nature of plant proteins. Free curcumin displayed multiple sharp peaks at 8.9°, 12.2°, 14.5°, 17.2°, 18.1°, 21.2°, 23.3°, 24.6°, 25.5°, and 27.3°, characteristic of its highly crystalline structure. These distinctive curcumin peaks were also visible in the physical mixtures (e.g., 8.9°, 12.2°, 14.5°, 17.2°, 21.2°), indicating that simple blending did not affect curcumin’s crystalline form. However, all PPI-Cur complexes lacked these characteristic curcumin reflections and instead showed only the amorphous protein halo, demonstrating that curcumin transitioned into an amorphous or molecularly dispersed state upon complexation. This complete disappearance of curcumin’s crystalline peaks suggests strong protein-curcumin interactions capable of preventing recrystallization (Yuan et al., 2021), consistent with the improved encapsulation performance of heat-treated samples—particularly H90—which provides a more favorable structural environment for curcumin dispersion.

### 3.9 Fluorescence quenching

Fluorescence quenching of proteins typically occurs through either dynamic quenching, arising from collisional encounters, or static quenching, resulting from ground-state complex formation between fluorophores and ligands. These mechanisms can be distinguished by examining the bimolecular quenching rate constant (Kq), as values of Kq significantly higher than the diffusion-controlled limit (∼2 × 10^10^ M^−1^·s^−1^) indicate static quenching (Kan et al., 2024). As shown in Table 3, heating markedly increased both Ksv and Kq, rising from 8.50 × 10^4^ M^−1^ and 8.50 × 10^12^ M^−1^·s^−1^ in NPPI to (2.38–2.52) × 10^5^ M^−1^ and (2.38–2.52) × 10^13^ M^−1^·s^−1^ in H80–H100. These high Kq values confirm that quenching occurred predominantly through a static mechanism, and that heating enhanced the formation of ground-state complexes between PPI and curcumin. Among all samples, H90 exhibited the highest Ksv and Kq, demonstrating the most favorable microenvironment for complex formation.

**Table 3:** Fluorescence quenching parameters for the interaction between curcumin and PPI before and after heat treatment.

Samples	Ksv (M^−1^)	Kq (M^−1^·s^−1^)	Ka (M^−1^)	n
NPPI	(8.50 ± 0.08) × 10^4c^	(8.50 ± 0.08) × 10^12c^	(8.05 ± 0.15) × 10^4c^	1.036 ± 0.007^c^
H80	(2.38 ± 0.01) × 10^5b^	(2.38 ± 0.01) × 10^13b^	(1.94 ± 0.02) × 10^5b^	1.101 ± 0.004^b^
H90	(2.52 ± 0.04) × 10^5a^	(2.52 ± 0.04) × 10^13a^	(2.00 ± 0.03) × 10^5a^	1.117 ± 0.003^a^
H100	(2.42 ± 0.01) × 10^5b^	(2.42 ± 0.01) × 10^13b^	(1.96 ± 0.01) × 10^5b^	1.101 ± 0.002^b^

Note: Values are presented as mean ± standard deviation. Different letters within the same column indicate significant differences (*p* < 0.05). Ksv: Stern–Volmer quenching constant; Kq: bimolecular quenching rate constant; Ka: binding constant; n: binding site number.

Consistently, the binding constant (Ka) increased from 8.05 × 10^4^ M^−1^ (NPPI) to (1.94–2.00) × 10^5^ M^−1^ in the heated samples, with H90 again achieving the highest affinity. This affinity level is comparable to or greater than those reported for other protein-curcumin systems—such as rice glutelin-curcumin (≈6.4 × 10^4^ M^−1^) (Li et al., 2024) and bovine serum albumin-curcumin complexes (≈2 × 10^5^ M^−1^) (Yang et al., 2013), indicating that heat-induced structural loosening in PPI markedly enhances ligand accessibility. A similar enhancement has been reported in β-lactoglobulin, where preheating increased the exposure of hydrophobic sites and significantly improved EGCG binding affinity (Shpigelman et al., 2010), indicating that moderate thermal unfolding facilitates stronger protein-polyphenol interactions. The binding site number (n) increased from 1.036 to 1.101–1.117 after heating, reflecting the exposure of additional accessible domains upon thermal loosening. Among the heated proteins, H90 consistently exhibited the highest Ksv, Ka, and n values, aligning with its greatest fluorescence red shift, highest surface hydrophobicity, and most favorable degree of unfolding. These features collectively facilitate stronger static binding with curcumin. In contrast, although H100 also showed enhanced binding relative to NPPI, its slightly lower affinity compared with H90 likely results from aggregation-induced shielding of potential binding sites.

### 3.10 Interfacial tension and interfacial rheology

**Fig. 4:**
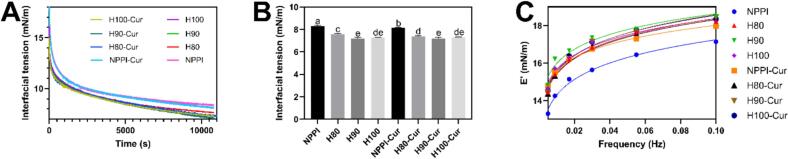
Interfacial tension profiles during 3 h adsorption (A), equilibrium interfacial tension after 3 h (B), and frequency-dependent dilatational elastic modulus of interfacial films formed by PPI samples and their curcumin complexes (C).

The interfacial tension profiles over 3 h and the equilibrium IT values are shown in Fig. 4A and B. NPPI exhibited the highest interfacial tension (8.31 ± 0.08 mN/m), indicating relatively weak interfacial activity due to its rigid conformation and limited hydrophobic exposure. Heat treatment significantly reduced interfacial tension, with H90 showing the lowest value (7.19 ± 0.10 mN/m), followed by H100 (7.30 ± 0.02 mN/m) and H80 (7.58 ± 0.08 mN/m). This enhancement is consistent with the heat-induced structural changes of PPI. Moderate heating promoted partial unfolding and increased surface hydrophobicity. Although some exposed hydrophobic groups were involved in intermolecular association, as indicated by the increased intermolecular β-sheet content, the heated proteins still exhibited higher surface hydrophobicity than NPPI. Moreover, H90 retained a relatively high absolute zeta potential, indicating good colloidal stability. These structural features together favored interfacial adsorption (Wang et al., 2012).

Curcumin incorporation exerted only minor effects on interfacial tension. NPPI-Cur exhibited a slight reduction in interfacial tension (8.14 ± 0.05 mN/m) compared to NPPI, suggesting modest improvement in interfacial adsorption due to curcumin-associated hydrophobic interactions. In contrast, H80-Cur, H90-Cur, and H100-Cur showed interfacial tension values nearly identical to their respective proteins, reflecting that heat-treated PPIs already possess highly exposed hydrophobic regions, leaving little room for additional enhancement upon curcumin loading. Notably, H90-Cur maintained the lowest interfacial tension, consistent with its optimal structural flexibility.

The frequency-dependent dilatational elasticity (E′) further revealed differences in interfacial film strength (Fig. 4C). All samples showed an increase in E′ with frequency, confirming the viscoelastic nature of protein-stabilized interfaces. Among the uncomplexed proteins, NPPI displayed the lowest E′, consistent with its limited unfolding and low surface hydrophobicity, which result in weaker interfacial film formation (Li et al., 2019). Heating enhanced interfacial elasticity, with H90 exhibiting the highest E′ across the entire frequency range, followed by H80 and H100, in agreement with their increased hydrophobic exposure.

Curcumin incorporation further modified the interfacial rheology. NPPI-Cur exhibited higher E′ values than NPPI, indicating that curcumin improved film cohesion, likely by promoting closer particle-particle packing during interfacial adsorption. In contrast, the heated complexes showed E′ values nearly identical to their corresponding proteins, consistent with the already optimized structural flexibility and strong intermolecular interactions of HPPI. Among all complexes, H90-Cur exhibited the highest overall elasticity, aligning with its most favorable balance between structural unfolding, surface charge, and strong protein-curcumin binding.

## 4 Conclusion

This study systematically investigated how thermal treatment (80–100 °C) alters the structural properties of pea protein isolate (PPI) and how these changes influence curcumin encapsulation and interfacial functionality. Heating between 80 and 100 °C progressively loosened the tertiary structure of PPI, and treatment at 90 °C created the most favorable configuration by enhancing hydrophobic exposure, increasing surface charge, and preventing excessive aggregation. These thermally induced structural adjustments improved the accessibility of binding sites, enabling curcumin to associate with PPI through a combination of hydrogen bonding and hydrophobic interactions. As a result, the 90 °C-treated protein achieved the highest encapsulation efficiency and formed small, uniform, and stable nanoparticles. These structural advantages translated directly to interfacial behavior. Both H90 and H90-Cur produced cohesive, elastic interfacial films that reflected more efficient adsorption and stronger interfacial network formation. The comparable performance of H90-Cur and H90 indicates that curcumin loading complements, rather than disrupts, the thermally optimized protein structure. Overall, the findings demonstrate that moderate thermal unfolding—specifically at 90 °C—effectively tunes PPI’s structural adaptability, strengthening its binding interactions with curcumin while enhancing nanoparticle assembly and interfacial stabilization. This work provides a clear structure-function framework for designing heat-modified plant proteins as efficient carriers for hydrophobic nutraceuticals and functional emulsifiers in food systems, and it may also support their application in beverage emulsions, functional dressings, and related nutraceutical delivery systems. However, this study mainly focused on structural characterization, encapsulation behavior, and interfacial functionality, while the biological activity, digestion behavior, and performance of the complexes in more complex food systems remain to be further investigated.

## CRediT authorship contribution statement

**Lijuan Luo:** Writing – original draft, Validation, Methodology, Formal analysis, Conceptualization. **Ping Li:** Conceptualization. **Hui Yang:** Supervision, Project administration, Funding acquisition. **Mingwei Zhang:** Writing – review & editing, Supervision.

## Declaration of competing interest

The authors declare that they have no known competing financial interests or personal relationships that could have appeared to influence the work reported in this paper.

## Data Availability

Data will be made available on request.
